# A retrieval-augmented chatbot based on GPT-4 provides appropriate differential diagnosis in gastrointestinal radiology: a proof of concept study

**DOI:** 10.1186/s41747-024-00457-x

**Published:** 2024-05-17

**Authors:** Stephan Rau, Alexander Rau, Johanna Nattenmüller, Anna Fink, Fabian Bamberg, Marco Reisert, Maximilian F. Russe

**Affiliations:** 1 Department of Diagnostic and Interventional Radiology, Faculty of Medicine, Medical Center – University of Freiburg, University of Freiburg, 79106 Freiburg Im Breisgau, Germany; 2 Department of Neuroradiology, Faculty of Medicine, Medical Center – University of Freiburg, University of Freiburg, Hugstetter Str. 55, 79106 Freiburg Im Breisgau, Germany

**Keywords:** Artificial intelligence, Diagnosis (differential), Gastrointestinal diseases, Knowledge acquisition (computer), Zero-shot learning

## Abstract

**Background:**

We investigated the potential of an imaging-aware GPT-4-based chatbot in providing diagnoses based on imaging descriptions of abdominal pathologies.

**Methods:**

Utilizing zero-shot learning via the LlamaIndex framework, GPT-4 was enhanced using the 96 documents from the Radiographics Top 10 Reading List on gastrointestinal imaging, creating a gastrointestinal imaging-aware chatbot (GIA-CB). To assess its diagnostic capability, 50 cases on a variety of abdominal pathologies were created, comprising radiological findings in fluoroscopy, MRI, and CT. We compared the GIA-CB to the generic GPT-4 chatbot (g-CB) in providing the primary and 2 additional differential diagnoses, using interpretations from senior-level radiologists as ground truth. The trustworthiness of the GIA-CB was evaluated by investigating the source documents as provided by the knowledge-retrieval mechanism. Mann–Whitney *U* test was employed.

**Results:**

The GIA-CB demonstrated a high capability to identify the most appropriate differential diagnosis in 39/50 cases (78%), significantly surpassing the g-CB in 27/50 cases (54%) (*p* = 0.006). Notably, the GIA-CB offered the primary differential in the top 3 differential diagnoses in 45/50 cases (90%) *versus* g-CB with 37/50 cases (74%) (*p* = 0.022) and always with appropriate explanations. The median response time was 29.8 s for GIA-CB and 15.7 s for g-CB, and the mean cost per case was $0.15 and $0.02, respectively.

**Conclusions:**

The GIA-CB not only provided an accurate diagnosis for gastrointestinal pathologies, but also direct access to source documents, providing insight into the decision-making process, a step towards trustworthy and explainable AI. Integrating context-specific data into AI models can support evidence-based clinical decision-making.

**Relevance statement:**

A context-aware GPT-4 chatbot demonstrates high accuracy in providing differential diagnoses based on imaging descriptions, surpassing the generic GPT-4. It provided formulated rationale and source excerpts supporting the diagnoses, thus enhancing trustworthy decision-support.

**Key points:**

• Knowledge retrieval enhances differential diagnoses in a gastrointestinal imaging-aware chatbot (GIA-CB).

• GIA-CB outperformed the generic counterpart, providing formulated rationale and source excerpts.

• GIA-CB has the potential to pave the way for AI-assisted decision support systems.

**Graphical Abstract:**

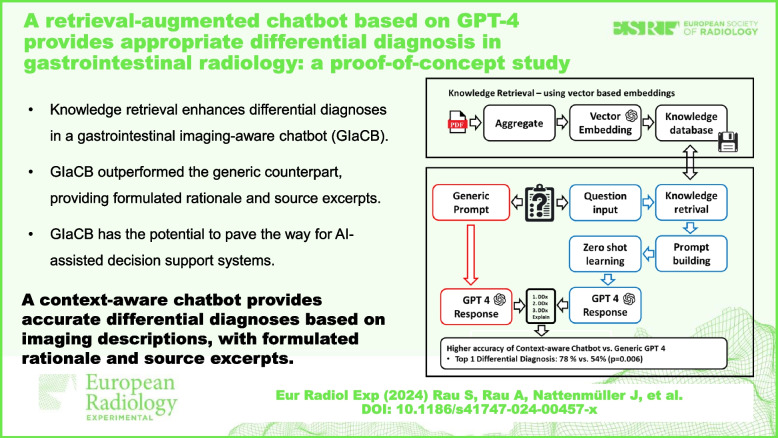

## Background

The increasing amount of imaging studies and the discovery of new pathological entities result in an increasing demand for radiologists’ expertise and time required for reporting [1]. Especially within the complex spectrum of abdominal pathologies, this poses challenges to clinicians due to overlapping and frequently unspecific symptoms [2–4]. Work-up of acute abdominal symptoms thus often requires imaging including fluoroscopy, computed tomography, and magnetic resonance imaging [5–7]. In addition, imaging plays an important role in differential diagnostics of neoplasia with an increasing complexity and number of entities and subtypes [8, 9].

Artificial intelligence (AI) and particularly models based on natural language processing (large language models [LLMs]) have the potential to streamline workflows and enhance accuracy while optimizing efficiency. The feasibility of integrating AI-based chatbots like OpenAI’s recently introduced Generative Pretrained Transformer 4 (GPT-4) [10] into the medical field was shown with GPT-4 providing clinical decision support on differential diagnoses and treatment and even passing the radiology board-style examination [11–13]. However, the transition into routine is hampered by the need for high-quality training data and the lack of transparency in the decision-making of LLMs [11, 14]. Additionally, GPT-4 is limited to its training data only including information until September 2021 [10]. Consequently, the latest body of research is lacking in the training data. Moreover, the exact training data remains elusive but includes not only scientific research but also text content that is not scientifically curated or reviewed openly, making it impossible to verify the content for a specific task and emphasizing the need for explainable AI [15].

By tailoring a LLM through integrating subject-specific content, reliable support in radiological clinical decision-making was shown [16, 17]. This modification of the LLM was achieved through so-called zero-shot learning and retrieval-augmented approaches, a method which allows a LLM to leverage their vast general knowledge together with a preselected specific database for the output, facilitating the model to address tasks, which they were not originally trained for [18]. Here, zero-shot learning introduced a specific knowledge (*i.e.*, a specific body of research required as background knowledge to fulfill the task) to the LLM. This is achieved by presenting the missing knowledge to the LLM during the query process as part of the prompt either manually or as part of a retrieval-augmented method automatically [19]. Zero-shot learning was first introduced in 2009 [20] and refined since 2020 by combining retrieval-augmented generation pipelines and transformers models [19]. While zero-shot learning is an established technique, the integration of zero-shot learning capabilities with GPT-4 represents a novel and evolving approach.

Despite the potential of AI-based decision-support, the trustworthiness is limited as insight into the decision-making process of the models is lacking [21]. Additional risks were also emphasized by the World Health Organization, particularly in the context of LLMs for medical diagnostics, which include hallucinations, data or automation bias, and skill degeneration of health care professionals [22]. In the case of context-aware chatbots or zero-shot learning, especially trustworthiness, hallucinations, and data bias could be addressed via context retrieval mechanisms, which reveal the specific text fragment from the tailored knowledge base on which the output is based.

This proof-of-concept study thus investigated the diagnostic potential of GPT-4 and a context-aware chatbot based on GPT-4 in terms of providing gastrointestinal differential diagnoses based on imaging descriptions of abdominal pathologies in a controlled setting with artificial case files comprising all information necessary to provide an imaging-based diagnosis.

## Methods

### Case compilation and ground truth

To assess the chatbots’ capability in providing differential diagnoses in gastrointestinal imaging, a compendium of fifty case files was prospectively created. These cases cover a broad range of gastrointestinal pathologies, sampled from the Radiographics Top 10 Reading List [23] comprising both common and rare entities. The 50 cases comprised a synthetic patient cohort (56% female, mean age 50.0 ± 14.4 years ranging from 7 months to 71 years) suffering from various abdominal pathologies, including malignancies (*n* = 17), inflammatory disorders (*n* = 15), obstructive disorders (*n* = 8), benign neoplasms (*n* = 6), vascular pathologies (*n* = 2), and other conditions (*n* = 2) (more details are provided in the supplementary material). Particular care was taken to ensure that the wording or content of the cases did not match the example cases from the articles. To resemble clinical routine workflow, each case file included a referral note with brief clinical context and information on age, sex, main complaint, and past medical history. Subsequently, the used imaging modality (fluoroscopy, MRI, or CT) and pathologic imaging findings were provided. Of note, the case files included the description of the radiological findings only, carefully ensuring the absence of leading diagnostic interpretations. The case descriptions, main differential diagnosis, and two additional differential diagnoses were established as ground truth through a combination of the used source documents and a consensus reading of two senior radiologists J.N. and M.F.R., who have 13 and 12 years of experience, respectively. This validation process was used to ensure the reliability of the comparative analysis and, moreover, to assure valid ground truth diagnoses.

### Technical implementation of the context-aware chatbot

As a knowledge database for creating the context-aware chatbot, the Radiographics Top 10 Reading List for gastrointestinal imaging was chosen. These 96 peer-reviewed documents serve as both a highly moderated and approved knowledge base and are an important educational resource for radiology trainees [23]. In order to handle this large dataset, we used the LlamaIndex framework (version 0.8.4) [24]. This is a dedicated, open-source Python library designed to facilitate retrieval-augmented approaches such as zero-shot learning for LLMs. The PDF files of the papers were imported and segmented sentence by sentence, and a numerical representation of the semantic content was generated using an embedding model by OpenAI (text-embedding-ada-002-v2) [25]. As metadata, and for later use as additional context, each of these sentences was stored along with the previous and following five sentences. As additional metadata, the name of each portable document format file and the page number of its content were retrieved. These texts, embeddings, and metadata were stored as a local vector store for reusability.

To extract the relevant content during the query, each case description was transformed into an embedding and was matched using a cosine similarity approach to the embeddings in the vector index. The fifteen best matches were extracted, and the texts were presented to the LLM. For this, OpenAI’s GPT-4 (version 06–13, released on 13 June 2023) was chosen, using backend access via the Application Programming Interface (API), rather than the general ChatGPT-frontend website [15]. The temperature parameter was set at a conservative value of 0.4, to moderate the probability distribution function of the model, influencing the level of creativity in its responses. To guide GPT-4 to answer in a robust style while incorporating the retrieved knowledge, a precision prompt with a structured template to state three differential diagnoses and an explanation was employed. The structure of the detailed prompt is as follows:

Prompt structure for GIA-CB:

*We have provided scientific context information below.*

{Top 15 text nodes retrieved from the Radiographics Top 10 Reading List on gastrointestinal imaging}

*Given this information, please solve the following case:*

{Clinical case information with imaging report}

*Please follow the structure below for your response as a list of differential diagnoses, only state the diagnosis, do not explain:*

- *Main differential diagnosis*
- *Second most likely differential diagnosis*
- *Third most likely differential diagnosis*

*Explanation: Provide a concise yet comprehensive and self-contained explanation summarizing the key points.*

Prompt for directly using GPT-4 by OpenAI (g-CB):

*Please solve the following case:*

{Clinical case information with imaging report}

*Please follow the structure below for your response as a list of differential diagnoses, only state the diagnosis, do not explain:*

- *Main differential diagnosis*
- *Second most likely differential diagnosis*
- *Third most likely differential diagnosis*

*Explanation: Provide a concise yet comprehensive and self-contained explanation summarizing the key points.*

To gain insight into the information that the retrieval approach considered important, the metadata, file name, and page were presented while creating hyperlinks to make the knowledge directly accessible to the radiologist. The resulting combination of vector store, automatic content retrieval, and GPT-4 by OpenAI using the prompting strategy creates the gastrointestinal imaging aware chatbot (GIA-CB).

To assess the performance of the retrieval-augmented approach in comparison with a generic chatbot (g-CB), we created a comparable setting using the pure GPT-4 and a similar precision prompt with the same structure and template while excluding the additional context. Costs of the models were captured for a subset of each five cases and the mean value is reported.

### Evaluation of the chatbots’ performance

The performances of the chatbots were evaluated based on the accuracy of the diagnoses using a three-tiered approach. First, we assessed whether the chatbot’s main differential diagnosis matched the main diagnosis in the ground truth. Second, to account for clinical variability, we investigated whether the chatbot’s main differential diagnosis was contained in the top three differentials in the ground truth. Finally, we assessed whether any of the chatbot’s top three differentials matched any of the correct ground truth diagnoses, assessing the chatbot’s ability to recognize the correct diagnosis within a set of plausible options. To compare the distributions of the two independent samples, *i.e.*, answers from GIA-CB and g-CB, the nonparametric Mann–Whitney *U* test was employed. Time per response was recorded and reported as a median with the interquartile range for each method; as it is dependent on OpenAI’s server performance, no further comparative tests were performed.

In cases where the diagnostic suggestions of the chatbots did not match the established ground truth, a descriptive analysis was performed through a consensus reading by experienced radiologists (J.N., M.F.R.). The diagnoses provided by the chatbot were examined for clinical soundness. The reasoning in the chatbot’s explanatory response was reviewed to assess whether the approach taken was in line with accepted clinical practice. For GIA-CB, the referenced sources were checked regarding appropriateness. When evaluating the results, it was taken into account that appropriate and accurate diagnostic procedures may lead to conclusions that do not strictly adhere to a predetermined list of differential diagnoses.

All statistical analyses were performed using the Python library SciPy (version 1.11.1). The significance level was set at *α* < 0.05 and the trend level at *α* < 0.10.

### Code availability

**Fig. 1:**
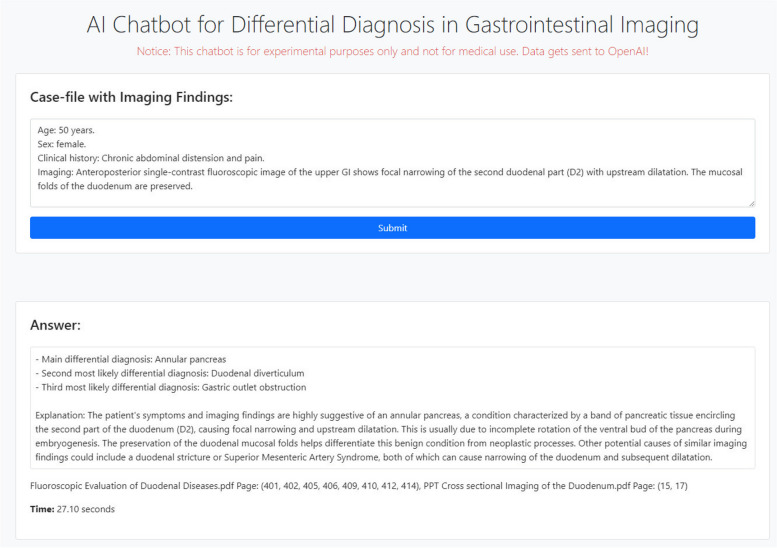
A screenshot of a web-based question–answer bot that simplifies case input and provides a clear presentation of results using case file #3 as an example

To demonstrate our context-aware chatbot implementation, we created a web-based question–answer bot that simplifies case input and provides a clear presentation of results (Fig. 1). The source code is publicly available on GitHub under the open-source MIT License (https://github.com/maxrusse/giaCB). The use of the code for research and other projects must be in accordance with the terms of the license.

## Results

### Statistical evaluation

There was a clear superiority in the performance of the contextualized GIA-CB over the counterpart g-CB as given in Table 1. In providing the main differential diagnosis and containing the main differential diagnosis within the top three differential diagnoses, giaCB performed significantly better than g-CB (*p* = 0.006 and *p* = 0.022, respectively). Regarding answers with at least one correct diagnosis according to the ground truth, a trend level difference was noted (*p* = 0.087). The median response time of the models was 29.8 s (IQR 4.34) for the GIA-CB and 15.7 s (IQR 4.31) for the g-CB. The mean cost per case was $0.15 for GIA-CB and $0.02 for g-CB.

**Table 1:** Accuracy of consistency to ground truth of main and differential diagnosis between the chatbots and ground truth

	Chatbot	Consistency to ground truth (ratio, percentage, 95% CI)
Main diagnosis matched	GIA-CB	39/50, 78% (0.67–0.96)
	g-CB	27/50, 54% (0.40–0.68)
Main diagnosis contained	GIA-CB	45/50, 90% (0.82–0.98)
	g-CB	37/50, 74% (0.62–0.86)
At least one fitting differential diagnosis	GIA-CB	49/50, 98% (0.94–1.00)
	g-CB	46/50, 92% (0.85–1.00)

*CI* Confidence interval, *GIA-CB* Gastrointestinal imaging-aware chatbot, *g-CB* Generic chatbot

### Assessment of diagnostic discrepancies in chatbot suggestions

In only one case (case #44), GIA-CB failed to provide the main diagnosis or at least one given differential diagnosis. The case reports a patient with nonspecific chronic abdominal symptoms and computed tomography findings of a mesenteric lesion with ill-defined margins and heterogeneous enhancement, indicating a sclerosing mesenteritis. Detailed case specifics can be found in the supplementary material. The GIA-CB falsely suggested mesenteric ischemia to be the most likely diagnosis followed by eosinophilic gastrointestinal disorders and small bowel entities. While the diagnosis is clearly off the mark, the assessment of the explanation showed that GIA-CB offers a detailed and precise explanation for the given differential diagnosis. Cross-checking the provided source documents by GIA-CB, the chatbot referenced paragraphs in the provided source documents matching the given differentials accordingly and summarized the specific information correctly. Moreover, the referenced sources from GIA-CB by the knowledge-retrieval mechanism were appropriate to the answers in all cases.

In an assessment of the five cases in which g-CB could not provide the most likely or the other given differential diagnoses, the generic GPT-4 tends to decide mainly on the basis of clinical information, guided by the symptoms, without taking the imaging findings fully into account. It is also apparent with respect to the answers in the further cases that the diagnoses are mostly formulated globally and only represent a directional indication instead of a precise diagnosis, *e.g.*, “pancreatic carcinoma” instead of “adenocarcinoma of the pancreas.” Similarly, the explanations are somewhat superficial. However, the given differential diagnoses are still close to the underlying pathology of the case. For example, in the case based on the main diagnosis of eosinophilic duodenitis, the case file described a patient with abdominal pain, altered bowel habits, and discomfort as well as computed tomography findings of duodenal wall thickening with mural stratification and a halo sign, as well as a mild affection of the jejunum and ascites (case #3) and g-CB suspected Crohn’s disease. As indicated above, this differential diagnosis fits the clinical picture, although it does not take the imaging findings into account.

## Discussion

Our findings underscore the potential capabilities of a context-aware chatbot based on GPT-4 by OpenAI to offer differential diagnoses based on imaging findings in the field of gastrointestinal diseases. In the evaluation of artificial radiological reports containing all information necessary for an imaging-based diagnosis, the context-aware GIA-CB demonstrated a robust capacity to find the most appropriate differential diagnosis in the majority of cases, achieving a high sensitivity and significantly surpassing the generic version. This could address the increasing demand for efficient and accurate diagnostic tools in radiology in light of the rapidly growing volume of diagnostic procedures [1, 14]. This is of special interest, as radiologists’ daily error rates were reported at 3–5% [26], whereas 9% of errors were reported to be due to wrongful attribution of image findings [27].

Previous studies on GPT-4 have shown remarkable potential in the medical field with promising applications [28]. For instance, GPT-4 was used to create summaries of radiological reports. In this setting, errors for complex and rare medical conditions were noted, but most likely due to insufficient sources of the model [13]. Furthermore, previous research highlighted ethical, legal, and data security considerations in AI-based solutions as they lack traceability and accountability [21, 29, 30].

These shortcomings can be addressed through innovative approaches based on zero-shot learning, where the model can understand and respond to tasks it has not explicitly been trained on. Integrating this approach with automatic knowledge retrieval, as in GIA-CB, can enrich the general knowledge of the LLM with a curated database. With this approach, the input to the LLM is not limited to a static prompt and the task (*e.g.*, a medical case file) but also integrates information from the database and then generates an answer based on the sources used. A transparent presentation of the sources increases traceability and supports accountability [18]. Promising experiences with zero-shot learning-based knowledge retrieval were already reported in the context of LLM-based decision-making for clinical imaging in accordance with ACR guidelines [17] and for identifying Arbeitsgemeinschaft Für Osteosynthesefragen − AO codes from radiology reports [16]. In contrast to the mentioned studies in which the knowledge base was segmented by an arbitrary number of tokens, we aimed to enhance the knowledge retrieval further through a fine granular sentence-based matching. This refined approach preserves sentence integrity, enabling precise identification of relevant document segments. Retrieval extends beyond the matching sentence to include previous and subsequent adjacent content. This mitigates edge case issues and includes information that may be just outside of sentence boundaries.

A previously published study by Ueda et al. [31] also evaluated the performance of the generic GPT-4 in the interpretation of radiological image findings. The LLM was prompted to find the correct solution based on the imaging findings from the “Diagnosis Please” quiz questions published in *Radiology*. The diagnostic performance using patient history and imaging findings was low at 61% and close to the performance of g-CB in our study with 54%, in contrast to our context-aware GIA-CB performed superior with 78% correct answers [32].

Nevertheless, the presented GIA-CB model does not work faultlessly. In the one case where GIA-CB was unable to provide a suitable differential diagnosis, although the model identified an incorrect pathology, the attached explanation and the source documents provided were consistent with the incorrect pathology presented. This indicates that the LLM based on GPT-4 itself performed adequately, while the knowledge retrieval mechanism provided insufficient semantic context, which probably led to the false assumption of the differential diagnosis. With the release of more advanced LLMs, it is expected that the amount of context that can be added to the prompt will continue to increase, allowing more sources to be included in knowledge retrieval [25]. Other more performance-intensive methods, such as graph-based knowledge, could also improve semantic adequacy [33].

In the future, a diagnostic tool following the example of the presented approach could contribute to an improved and efficient diagnostic workup and a case-based training tool. Moreover, with improved image analysis tools, automatic report generation independent of radiological reporting is conceivable. Nevertheless, the results of this study indicate that a human control instance will remain essential.

The performance of the proposed approach when integrated into the clinical workflow and comparison with human diagnostic performance remains to be evaluated. Following research could also investigate the influence of such tools on the diagnostic confidence of radiologists and the potential to optimize time efficiencies. Of note, both models calculated the results per case in less than a minute. Further studies should also evaluate the potential of knowledge retrieval integrated into other LLMs like Gemini, the recently introduced update of the LLM Bard by Google which showed promising high-performance characteristics in its ability to process and interpret complex data sets [34].

In addition, the topic-specific reference used for this study only provides a good overview of the field of gastrointestinal pathologies, some rare pathologies might not be covered and therefore are not identifiable by the proposed approach. A dynamic extension of the index is possible in this respect, and a more comprehensive compendium, *e.g.*, moderated by the subspecialty society, would be desirable.

Though the initial results are promising, the generalizability of the proposed approach on real-world data (*e.g.*, including typing errors, missing imaging signs, incomplete medical history) has yet to be confirmed. Moreover, further studies should focus on the added value of a context-aware chatbot in the circumstance that a human reader correctly identified all imaging features but is lacking an appropriate imaging-based diagnosis. A potential automation bias (*i.e.*, unnecessary confusion of the involved radiologists and referring clinicians) is conceivable. However, revealing the source from the underlying knowledge base allows for a quick access to the literature, the chatbot deemed important for human double-checking. While this provides a certain insight into the text information the model relies its output on, the decision-making process itself remains not transparent.

Eventually, potential applications of the presented diagnostic support tool may comprise the integration into reporting structures. Here, it could offer automated suggestions for differential diagnoses in the assessment of radiological reports. Furthermore, the tool could facilitate quality assurance, providing secondary, retrospective analysis of radiological findings to assess and ensure diagnostic accuracy across centers.

In summary, the results from this proof-of-concept study indicate that context-aware GPT-4-based algorithms have the ability to accurately offer differential diagnoses based on radiology image findings. By granting insight into the consulted source documents, it supports transparency and trustworthiness in the decision-making process, enhancing its auditability. Such a tool may improve reliable and evidence-based differential diagnostics.

## Data Availability

The datasets used and analyzed during the current study are available from the corresponding author upon reasonable request. The code generated during the current study is available in the “GitHub” repository, https://github.com/maxrusse/giaCB.

## Abbreviations

AI: Artificial intelligence
g-CB: Generic chatbot
GIA-CB: Gastrointestinal imaging-aware chatbot
GPT-4: Generative Pretrained Transformer 4
LLM: Large language model
